# Acute worsening of clinical presentation in CRPS after SARS-CoV-2 (COVID-19) vaccination: a case series

**DOI:** 10.2217/pmt-2021-0089

**Published:** 2021-11-23

**Authors:** Jessica Zhang, Semih Gungor

**Affiliations:** ^1^Research Assistant, Department of Anesthesiology, Critical Care & Pain Management, Hospital for Special Surgery, New York, NY 10021, USA; ^2^Attending Physician, Department of Anesthesiology, Critical Care & Pain Management, Hospital for Special Surgery, New York, NY 10021, USA; ^3^Assistant Professor of Anesthesiology, Department of Anesthesiology, Weill Cornell Medicine, New York, NY 10065, USA

**Keywords:** coronavirus, COVID-19, CRPS, mRNA, pain, RSD, vaccination

## Abstract

We present the acute worsening of clinical presentation in complex regional pain syndrome (CRPS), following mRNA-based COVID-19 vaccination. We report the case series of three patients diagnosed with CRPS type I who presented with acute worsening of symptoms after mRNA-based COVID-19 vaccination and their medical management. The acute clinical worsening after mRNA-based COVID-19 vaccination was transient and effectively managed with adjustment of individualized therapy in all three patients. The mortality and morbidity of COVID-19 infection are serious, and vaccination is recommended in the general population, including patients with a diagnosis of CRPS. However, clinicians should be aware of the possibility that there may be a clinical worsening CRPS after mRNA-based COVID-19 vaccination.

## Lay abstract

This case series presents the worsening of symptoms in complex regional pain syndrome (CRPS), following mRNA-based COVID-19 vaccination. We report on three patients diagnosed with CRPS who presented with worsening of symptoms after mRNA-based COVID-19 vaccination and their medical management. The worsening of symptoms was temporary and managed with adjustment of individualized therapy in all three patients. The dangers of COVID-19 infection are serious, and vaccination is highly recommended in the general population, including patients with a diagnosis of CRPS. However, healthcare providers should be aware of the possibility of clinical worsening of CRPS after mRNA-based COVID-19 vaccination.

**Practice points**

- Complex regional pain syndrome (CRPS) has unknown causes, but there are speculations of an autoimmune etiology.
- Exacerbation of clinical presentation of CRPS occurred in three patients after receiving mRNA-based coronavirus disease 2019 vaccination.
- Although there have been past suggestions of the association of vaccines, particularly with the human papillomavirus vaccine, and development of CRPS, further investigations concluded no such causal relationship.
- Diphenhydramine is shown to be a potential treatment in reducing CRPS symptoms as it was successfully utilized for one of the three cases.
- Other treatments used included gabapentin, lumbar sympathetic block, lorazepam, baclofen and hydromorphone.
- Each case of exacerbation had different treatment methods, illustrating the difficulty in treating CRPS.
- One of the three cases also experienced worsening of CRPS symptoms after the second vaccine dose, whereas the other two cases only experienced it with the first dose.
- One patient who had preventive treatment prior to the second vaccine dose did not experience worsening of symptoms.
- Increasing current medication dosage or introducing new medication before vaccination are possible preventative measures.

Severe acute respiratory syndrome coronavirus 2 (SARS-CoV-2), also known as COVID-19, has led to an acute respiratory disease pandemic, resulting in global morbidity and mortality [1].

The mRNA technology is an emerging platform for vaccine development. Two COVID-19 vaccines were developed using this new technology, and the US FDA granted emergency authorization for use in the USA [2,3]. The reported side effects of vaccines developed using mRNA technology have been mild and self-limited [2].

Complex regional pain syndrome (CRPS) is a chronic pain condition characterized by a collection of sensory, motor and autonomic changes that usually follow trauma or autoimmune stress. The pathophysiology of CRPS is not well known, but research suggests CNS inflammation with glial cell activation in the spinal cord and brain. Others have hypothesized that CRPS relates to peripheral neurogenic inflammation and autoimmunity [4]. Because of the complexity of pathophysiology, CRPS is challenging to treat and requires further examination.

We present a case series of three patients diagnosed with CRPS who experienced a moderate to severe worsening of symptoms and signs following an mRNA-based COVID-19 vaccination. This research study was approved by the hospital's Institutional Review Board and written informed consent was obtained by all patients for the publication of this research.

## Case series

### Case 1

The patient is a 23-year-old otherwise healthy female with a history of right lower extremity CRPS type I. The patient underwent a series of lumbar sympathetic blocks and physical therapy for 1 year with over 90% improvement of the right ankle and foot pain and symptoms. The patient could function well, and she did not require any medication except over-the-counter non-steroidal anti-inflammatory drugs (NSAIDs) and acetaminophen. The patient developed a severe exacerbation of the burning pain in the right foot extending proximally to the entire right lower extremity that started 3 h after receiving the first dose of mRNA-based COVID-19 vaccination (BNT162b2 COVID-19, Pfizer/BioNTech). The patient did not report any new trauma. The patient developed sensitivity to touch, swelling, color changes and temperature changes in the right leg, ankle and foot. Moreover, the patient developed intense lower extremity myoclonus in the entire right lower extremity. The patient was treated in the emergency room with intravenous (IV) ketorolac 30 mg, hydromorphone and diazepam with the minimal improvement of the pain and symptoms. The patient was admitted to the hospital for further management. The patient was treated in the intensive care unit, and medications including IV ketamine infusion with the dose titrated up to 20 mg/h, IV hydromorphone patient-controlled analgesia, IV diazepam 5 mg as needed for muscle spasms and diphenhydramine (DPH) 25–50 mg IV every 8 h as needed for muscle spasms were administered. Although there was a partial improvement of the right lower extremity pain and myoclonus, the patient was still complaining of intermittent severe muscle spasms approximately every 4 h. The patient was started on oral lorazepam 2 mg every 6 h as needed, oral baclofen 10 mg orally every 8 h for muscle spasms, DPH 25–50 mg orally every 8 h as needed for muscle spasms and oral hydromorphone 2–6 mg orally every 3 h as needed. The patient underwent physical therapy twice a day while she was at the hospital. The patient underwent a right-sided lumbar sympathetic block under fluoroscopic guidance on the second day of the admission due to persistent pain and spasms. The patient reported over 70% improvement of the pain and symptoms after the procedure. IV ketamine was weaned off within 1 day after the sympathetic block, and IV patient-controlled analgesia was weaned off over 2 days, and the patient was transferred to the regular inpatient room. The patient was discharged home on the sixth day of admission with medications including hydromorphone 2–4 mg orally every 4 h as needed, baclofen 10 mg orally every 8 h, lorazepam 1–2 mg orally every 8 h as needed for muscle spasms and DPH 25–50 mg orally every 8 h for muscle spasms. The patient could wean all the medications within 10 days after discharge from the hospital with the improvement of the right lower extremity pain and symptoms to baseline requiring only over-the-counter naproxen and acetaminophen as needed.

Prior to the second dose of COVID-19 vaccination, 3 weeks after the first dose, it was decided to treat the patient prevaccination with the anticipation that there might be a worsening of the symptoms again. The patient underwent lumbar sympathetic block 2 days before the second dose of COVID-19 vaccination (BNT162b2 COVID-19, Pfizer/BioNTech) and was started on DPH 50 mg orally every 8 h around the clock and 48 h before the second dose of vaccination.

The patient did not report any significant worsening of the right lower extremity pain and symptoms after the second vaccination dose. The patient followed up with the provider for 5 months after the second dose, and there was no significant worsening of the CRPS-related pain and symptoms. The patient was complaining of mild baseline pain and symptoms, requiring over-the-counter naproxen and acetaminophen as needed.

### Case 2

The patient is a 21-year-old otherwise healthy male diagnosed with bilateral lower extremity CRPS type I, 2 years ago. The patient completed physical therapy, underwent a series of bilateral lumbar sympathetic blocks and was maintained on Gabapentin 400 mg in the morning, 600 mg in the afternoon and 600 mg at night time. The patient was stable on this dose with mild baseline pain without interfering with daily activities. The patient underwent the first dose of mRNA COVID-19 vaccination (mRNA-1273 vaccine, Moderna) and reported severe exacerbation of bilateral lower extremity burning pain within 24 h after the first dose. The patient was consulted on the phone, and the dose of gabapentin was increased to 600 mg in the morning and 600 mg in the afternoon and 800 mg at night-time. The patient did not require any additional medication for pain control. Approximately 2 weeks after the first vaccination dose, the patient could wean to the baseline dose of gabapentin. After the second dose of COVID-19 vaccination (mRNA-1273 vaccine, Moderna), 4 weeks after the first dose, the patient again experienced worsening bilateral lower extremity burning pain with intermittent color and temperature changes. Furthermore, the dose of gabapentin was increased to 600 mg in the morning, 600 mg in the afternoon and 800 mg at night-time to improve the pain control. The patient could wean gabapentin successfully back to baseline after 3 weeks, following the second dose of vaccination. The patient was followed up for 4 months after the second dose of vaccination. The patient did not require any additional dose of pain medication and was stable with the mild pain on the baseline dose of gabapentin. The patient did not require any additional pain medication or intervention.

### Case 3

The patient is a 49-year-old female with a history of right lower extremity CRPS type I, diagnosed 3 years ago. The patient’s other medical history is significant for migraine headaches, for which she has been on topiramate 200 mg daily dose.

The patient underwent a series of intermittent lumbar sympathetic blocks, physical therapy, medication management, activity modification, home exercises and underwent dorsal root ganglion neurostimulator implantation, following a successful trial 18 months ago. The patient reported moderate pain control with all of these treatment modalities. She was on pregabalin 75 mg orally twice a day for neuropathic pain and indomethacin 75 mg twice a day as needed.

The patient could function and work despite the requirement for ice application to the right lower extremity, which seems to relieve the burning sensation from time to time. The patient reported severe worsening of the right lower extremity, burning pain, color changes and temperature changes within 24 h after the first dose of mRNA COVID-19 vaccination (mRNA-1273 vaccine, Moderna). The patient was consulted over the phone, and the dose of pregabalin was increased to 75 mg every 8 h. As there was still significant pain the next day, the patient was started on duloxetine 20 mg daily dose, which was gradually increased to 60 mg daily dose over 3 weeks duration. The patient continued with indomethacin 75 mg twice a day with a more frequent ice pack application to the lower extremity for pain relief.

The patient received a second dose of COVID-19 vaccination (mRNA-1273 vaccine, Moderna), 4 weeks after the first dose, and she did not report any significant worsening of the CRPS-related pain and symptoms after the second dose. The patient could wean pregabalin to 75 mg twice a day and stayed on duloxetine 60 mg daily and indomethacin 75 mg twice a day as needed 2 months after the second dose of vaccination.

## Discussion

CRPS is characterized by pain with a wide range of symptoms affecting one or more body limbs. CRPS can be challenging to manage because the pathophysiology is unclear [5]. Proposed sources of inflammation in CRPS involve neuroinflammation, neurogenic inflammation, dysregulation of the immune system and predominantly peripheral neurogenic inflammation [4,6].

The relation between CRPS and autoimmunity has been explored in the past research. The presence of active autoantibodies (AAb) against the β2 adrenergic receptors (AdR) and muscarinic-2 (M2) receptors has been shown in the sera of the patients with the diagnosis of CRPS, supporting the concept of an autoimmune etiology [5]. There are also studies showing the presence of significantly higher autoantibodies and antinuclear antibodies in a subset of patients with CRPS 1 [7]. Moreover, antineuronal antibodies have also been associated with CRPS in 30–40% of the patients [8]. Although the study found autoantibodies against differentiated autonomic neurons in patients with CRPS, the role that the antibodies play in the development, severity and exacerbation of CRPS is incompletely understood.

The relation between CRPS and vaccines has been examined in the past. Ozawa *et al.* reported a temporal relationship between the human papillomavirus (HPV) vaccine administration and CRPS [9]. Further investigations evaluated the causal relationship between HPV vaccines and CRPS along with other autonomic conditions and determined there was no statistically significant evidence of such a relationship and that the observed CRPS incidences was less than expected [10,11]. Although there was no direct causal relationship delineated between the HPV vaccine and CRPS development, the potential association between vaccines and exacerbating CRPS cannot be ruled out.

Vaccines have also been associated with inflammatory conditions through adjuvants, also known as autoimmune or inflammatory syndrome induced by adjuvants. Adjuvants are components that enhance the immune response to specific antigens and include but are not limited to silica, aluminum hydroxide and squalene [12].

The activation of α1 AdR may cause CRPS pain and dysautonomia symptoms, while the activation of β2 AdR and M2 anticholinergic receptors (AChRs) may participate in the modulation of pain and inflammation response. In the majority of CRPS patients, anti-α1 AdR AAb, anti-β2 AdR AAb and anti-M2 AChR AAb showed receptor agonist activity in functional assays [13].

The mRNA vaccine is administered as the intramuscular injection of two separate doses of a nucleoside-modified messenger RNA (modRNA) that encodes for the viral spike (S) glycoprotein of SARS-CoV-2 [2,3]. Vaccines work by eliciting an immune response by producing antibodies against the substances in the vaccine, modRNA of the S antigen in the mRNA COVID-19 vaccines [2,3,14].

While no serious neurologic or neuroinflammatory adverse event patterns were reported in clinical trials of the mRNA-1273 vaccine (Moderna), 10.1% of vaccine trial participants experienced muscle pain preventing daily activity after the second dose compared with the 0.4% of the placebo group [3].

Therefore, the autoimmune origins of CRPS may be one of the proposed mechanisms by which the mRNA-based COVID-19 vaccination causing increased autoimmune response could exacerbate symptoms of CRPS in this case series of three patients.

We would like to point out that in Case 1, the clinical presentation was severe and included involuntary muscle spasms in the affected extremity. Motor symptoms and signs are described in approximately 25% of the cases with CRPS and are often resistant to treatment [15]. In this case, we successfully used DPH for the treatment of muscle spasms.

DPH has been documented as a successful treatment for controlling extrapyramidal symptoms associated with CRPS. While the mechanism of action is unclear, it has been hypothesized that DPH’s anticholinergic and antihistaminic effects are associated with the beneficial results of reducing extrapyramidal signs [16].

The patients had experienced no other recent changes or events that could have led to the observed symptoms besides having had the vaccine. Prior to these incidences, all three cases had undergone physical therapy and bilateral lumbar sympathetic blocks, and Cases 2 and 3 were on medication regimen. All three cases experienced worsening of CRPS syndromes within 24 h of the first mRNA-based vaccine dose, but only Case 2 reported significant worsening of CRPS after the second vaccine dose. Case 1 was treated 2 days prevaccination with lumbar sympathetic block, whereas the other two cases did not. Case 2 did not need to introduce new medications for symptom relief, but Case 3 had to add duloxetine and Case 1 was given multiple medications for pain relief and underwent a lumbar sympathetic block which improved response to the second vaccine dose. The responses to these interventions suggest that providing treatment before vaccine administration may prevent worsening of CRPS symptoms. Prevention measures can include increasing dosage of current medication or introducing a new medication, especially for those who may not have been taking medication regularly such as the patient in Case 1. Despite similar worsening of symptoms of CRPS within 24 h of vaccine administration, each case was treated individually and tailored to their already existing pain control routine. As the worsening of the symptoms was only in a small subset of patients with a diagnosis of CRPS after COVID-19 vaccination, we recommend counseling and closely monitoring these patients after vaccination and being ready to institute individual treatment if necessary. However, if a patient presents with the worsening of symptoms after a dose of COVID-19 vaccination, we recommend prophylactic and individualized treatment such as increasing the dose of medications or performing interventions prior to the next vaccination doses.

## Conclusion

The mortality and morbidity of COVID-19 infection are serious, and vaccination is highly recommended in the general population, including patients diagnosed with CRPS. However, the clinicians should be aware of the possibility that there may be a clinical worsening of CRPS after mRNA-based COVID-19 vaccination. In our case series, clinical worsening was transient and effectively managed with adjustment of individualized therapy.
